# A rapid method for isolation of bacterial extracellular vesicles from culture media using epsilon-poly-L–lysine that enables immunological function research

**DOI:** 10.3389/fimmu.2022.930510

**Published:** 2022-08-12

**Authors:** Shujin Wei, Dian Jiao, Wanli Xing

**Affiliations:** ^1^ School of Medicine, Tsinghua University, Beijing, China; ^2^ School of Life Sciences, Tsinghua University, Beijing, China

**Keywords:** bacterial extracellular vesicles, epsilon-poly-L–lysine, bacterial culture medium, isolation, precipitation, innate immunity, cytokine

## Abstract

Both Gram-negative and Gram-positive bacteria can release vesicle-like structures referred to as bacterial extracellular vesicles (BEVs), which contain various bioactive compounds. BEVs play important roles in the microbial community interactions and host-microbe interactions. Markedly, BEVs can be delivered to host cells, thus modulating the development and function of the innate immune system. To clarify the compositions and biological functions of BEVs, we need to collect these vesicles with high purity and bioactivity. Here we propose an isolation strategy based on a broad-spectrum antimicrobial epsilon-poly-L-lysine (ϵ-PL) to precipitate BEVs at a relatively low centrifugal speed (10,000 × g). Compared to the standard ultracentrifugation strategy, our method can enrich BEVs from large volumes of media inexpensively and rapidly. The precipitated BEVs can be recovered by adjusting the pH and ionic strength of the media, followed by an ultrafiltration step to remove ϵ-PL and achieve buffer exchange. The morphology, size, and protein composition of the ϵ-PL-precipitated BEVs are comparable to those purified by ultracentrifugation. Moreover, ϵ-PL-precipitated BEVs retained the biological activity as observed by confocal microscopy studies. And THP-1 cells stimulated with these BEVs undergo marked reprogramming of their transcriptome. KEGG analysis of the differentially expressed genes showed that the signal pathways of cellular inflammatory response were significantly activated. Taken together, we provide a new method to rapidly enrich BEVs with high purity and bioactivity, which has the potential to be applied to BEVs-related immune response studies.

## Introduction

One of the functions of the innate immune system is to surveil the microbes in our bodies. Innate immune cells can recognize conserved bacterial molecular structures known as pathogen-associated molecular patterns (PAMPs) through pattern-recognition receptors (PRRs), which are crucial for the development of appropriate immune responses (1, 2). Even though the access of microbes to the immune cells is restricted physically in most situations, recent evidence have shown that the microbiota can communicate with the host through various effector molecules (3), such as short-chain fatty acids (SCFAs), lipopolysaccharide (LPS), proteins, and bacterial extracellular vesicles (BEVs) (4). Among these microbiota-secreted factors, BEV is likely to have a more important role in interkingdom interactions (5–7) for the release of vesicle-like structures is a universally conserved cellular process that occurs in all domains of life (8). BEVs harbor various components derived from bacterial cells, like genetic materials, proteins, lipids, and virulence factors. By interacting with innate immune cells, BEV can regulate immune reactions in the host (9, 10). Evaluation of the interaction between BEV and the innate immune system can provide a better understanding of the molecular mechanisms underlying innate immune responses, and has the potential to develop a new avenue of therapies on the basis of BEVs.

Unfortunately, the isolation of BEVs is still facing some challenges. Similar to exosomes secreted by mammal cells, BEVs are heterogeneous vesicles and have quite small diameters in the range from 20 to 200 nm (11, 12). Until now, the most commonly used BEV enrichment method is ultracentrifugation (> 100,000 × g) (13, 14), which requires expensive instrumentation, long processing times, and cumbersome operation. Other available enrichment methods include ultrafiltration (UF), precipitation by addition of a high concentration of salt, and gel filtration (15). However, each of these methods suffers from its disadvantages (16). In UF techniques, the membrane is easily clogged, retarding the process of isolation. In precipitation techniques, the concentration of salt should be raised to more than 40%, and the non-specific binding of free proteins to BEV is highly probable. As to gel filtration technique, many factors, including column packing, flow rate, media types, and pore size should be considered to achieve high efficiency. There is an urgent need to develop more convenient methods for BEV isolation.

Some features of BEVs can be used to develop new isolation methods. It has been shown that BEVs derived from Gram-negative bacteria harbor abundant amounts of LPS on the membrane (17, 18), while lipoteichoic acid is often considered a component of Gram-positive bacterial extracellular vesicles (19). Hence the BEVs often have a concentrated negative charge. ϵ-Poly- L -lysine (ϵ -PL) is a natural antimicrobial substance and consists of 25 to30 L-lysine residues which possess positively charged amine groups (20). ϵ-PL has broad-spectrum antimicrobial activity and low toxicity; hence it is utilized as a food additive for various products. The interaction of bacteria and ϵ-PL relies on negative charges on the bacterial membrane (21, 22). Nanoparticles functionalized with ϵ-PL have been used for broad-spectrum bacterial capture (23). Yet there’s no report applying this substance in BEVs enrichment.

In this work, we established an ϵ-PL-based technique to enrich BEVs from bacterial culture media at a relatively low centrifugal speed (10,000 × g). The extracted materials isolated by our new method have similar protein profiles as the BEVs isolated by ultracentrifugation. Subsequently, by adjusting the pH and ionic strength of the buffer, the pellets could become dispersed in suspension without visible aggregation. We then conducted ultrafiltration to remove ϵ-PL and achieve buffer exchange. The morphology and size of the harvested BEVs were found to be comparable to those purified by ultracentrifugation. Finally, we validated that the ϵ-PL-precipitated BEVs can be internalized by THP-1 cells and induce activation of inflammation-related signaling pathways. Overall, it suggested that our method could potentially contribute to studies on BEVs and their immunoregulatory effect on innate immunity.

## Materials and methods

### Microbial culture and preparation of culture medium

*Escherichia coli* laboratory strain DH5a (*E. coli*) and *Staphylococcus aureus* (*S. aureus*, CICC 10384) were grown overnight in LB broth (1% tryptone, 0.5% yeast extract, 1% NaCl) at 37°C with shaking (200 rpm). The supernatant fraction was collected by centrifugation (6,000 g, 15 min, 4°C, and 10,000 g, 15 min, 4°C). Then the supernatant was filtered through a PVDF 0.45-µm filter (HYCX, China) to remove any remaining cells. The resultant filtrate could be used for subsequent BEV isolation.

### Isolation of BEVs by ultracentrifugation

The bacterial culture medium preprocessed by centrifugation and filtration was loaded into ultracentrifuge tubes and centrifuged at 160,000 × g for 2h at 4°C (rotor 70Ti, L-80XP, Beckman Coulter, Germany) to obtain vesicle-rich pellets. Then the pellets were resuspended in phosphate buffer saline (PBS) and centrifuged at 160,000 × g for 2h at 4°C. Finally, the pellets were resuspended in a minimal volume of PBS and stored at −80°C until use.

### Isolation of BEVs by ϵ-PL-based method

3 M 4-Morpholineethanesulfonic acid (Aladdin, China) was combined with sodium chloride (0.15 M) to make a ten-fold concentrated (10× MES) stock solution. The 10x MES solution was added to a tenfold volume of bacterial culture medium preprocessed by centrifugation and filtration to adjust the pH value of the bacterial culture medium to near neutrality. The **ϵ**-PL (Shanghai yuanye, China) stock solution was prepared by dissolving **ϵ**-PL in PBS at a concentration of 10 mg/mL. Then the **ϵ**-PL stock solution was added to the bacterial culture medium to achieve a final concentration of 100 μg/mL. After rocking on a shaker for 45 min at room temperature, the mixture was subjected to centrifugation at 10,000 × g for 15 min. Then the pellet was washed in PBS buffer twice and resuspended by re-suspension buffer (50 mM Tris-HCl, 0.5 M NaCl, pH 8.5). Finally, the sample was subjected to four rounds of ultrafiltration using 1.5-mL 100 kDa ultrafiltration tubes (Merck Millipore, Germany). The buffer was replaced by PBS and stored at −80°C until use.

### Transmission electron microscopy

BEVs isolated by UC or ϵ-PL were diluted to an appropriate concentration. Then, 7 μL of the BEVs were dropped onto carbon-coated grids and incubated for 2 min at room temperature. Next, BEVs were negatively stained with uranyl acetate for 1 min. Finally, BEVs were observed using an electron microscope (FEI Tecnai Spirit TEM D1266, FEI, USA) operating at 110 kV.

### Nanoparticle tracking analysis

BEVs isolated by UC or ϵ-PL were diluted 3000-fold with distilled water. The particle size distribution of BEVs was determined using nanoparticle tracking analysis (Particle Metrix, Germany). All particle-size analyses used the same set of parameters to ensure comparable results.

### Bicinchoninic acid assay

The BEV protein concentration was quantified using the BCA protein assay kit (Solarbio, China) following the manufacturer’s instructions.

### Proteomic analysis of BEVs by LC-MS/MS

BEVs isolated by UC or ϵ-PL were used for proteomic analysis. BEVs (10 μg) were separated by SDS-PAGE (10%). Then the gel bands were excised and subjected to in-gel digestion. The peptides were extracted redissolved in 0.1% TFA solution and analyzed by LC-MS/MS.

For LC-MS/MS analysis, the peptides were separated by a 120 min gradient elution at a flow rate of 0.30 µL/min with a Thermo-Dionex Ultimate 3000 HPLC system, which was directly interfaced with a Thermo Scientific Q Exactive™ HF-X mass spectrometer. The mass spectrometer was operated in the data-dependent acquisition mode using Xcalibur 2.2 software and there was a single full-scan mass spectrum in the orbitrap (300-1800 m/z, 60,000 resolution) followed by data-dependent MS/MS scans at NCE 27%.

The MS/MS spectra from each LC-MS/MS run were searched against datasets from UniProt using an in-house Proteome Discoverer (Version PD1.4, Thermo-Fisher Scientific, USA). The peptide false discovery rate (FDR) was calculated using Percolator provided by PD. The peptide spectrum match (PSM) was considered to be correct only when the q-value was smaller than 1%. FDR was determined based on PSMs when searched against the reverse, decoy database. Peptides only assigned to a given protein group were considered unique. The false discovery rate (FDR) was set to 0.01 for protein identification.

Analysis of proteins was performed as previously described (24, 25). Spectral counts of each protein are normalized for quantification. Statistical testing was performed using R software 4.0.2 (R Foundation for Statistical Computing, Austria) (26).

### Cytotoxicity assessment

THP-1 monocytes were seeded in 96-well plates (4 × 10^4^ cells per well) and let grow for 24h under 5% CO_2_ at 37°C. The cells were then treated with different protein concentrations (5, 10, and 15μg/ml) of BEVs for 6 h. PBS-treated cells were considered a negative control with 100% viability. The viability assay was conducted using the Cell Counting Kit-8 (CCK-8) assay kit (Lablead, China) following the manufacturer’s instructions.

### Assessment of uptake of BEVs

Analysis of the internalization of BEVs was performed as previously described (27). Briefly, BEVs were diluted in 500 μL PBS, and 2 μL DiI dye (Thermo Fisher Scientific, USA) was added and incubated for 30 min at 37°C. After incubation, the samples were transferred to 1.5-mL 100-kDa ultrafiltration tubes and washed four times with PBS at 14,000 × g. The filters were inverted and centrifuged at 1000 × g for 2 min to collect the labeled BEVs. PBS labeled with DiI using the same protocol performed with BEVs was used as the control for uptake study.

THP-1 cells were seeded in 96-well plates (2 × 10^5^ cells per well). Then labeled BEVs were added onto cells at protein concentrations of 5, 10, and 20 μg/mL and incubated for 4h under 5% CO_2_ at 37°C. The cells were washed three times with PBS to remove residual BEVs. The cellular suspensions were collected and measured with flow cytometry. DiI-positive cells (PE-A channel) after successful uptake of DiI-labeled BEVs were determined, as compared with PBS-treated cells (negative control), using FlowJo 7.6 software (FlowJo LLC, USA).

To conduct confocal imaging of cells, THP-1 cells incubated with BEVs at a protein concentration of 20 μg/mL were fixed with 4% paraformaldehyde for 15 min and stained with 4’, 6-diamidino-2-phenylindole (DAPI, Thermo Fisher Scientific, USA) for 3 min. The cells were analyzed with a confocal laser scanning microscope (Zeiss, Germany).

### Transcriptional analysis of THP-1 cells stimulated with BEVs

THP-1 monocytes were seeded at a density of 0.75 × 10^6^ cells per mL in 6-well plates in 2 mL RPMI 1640 medium (Thermo Fisher Scientific, USA) supplemented with 10% fetal bovine serum (Gibco, USA) and antibiotics. Then *E. coli* BEVs at a protein concentration of 1μg/mL and *S.aureus* BEVs at a concentration of 10 μg/mL were added to each well followed by incubation for 6 h. Cells stimulated with PBS served as the negative control. The experiments were performed in triplicate for each group.

Total RNA was extracted with Trizol (Invitrogen) and assessed with Agilent 2100 BioAnalyzer (Agilent Technologies, Santa Clara, CA, USA) and Qubit Fluorometer (Invitrogen). The NEB Next Ultra RNA Library Prep Kit for Illumina (NEB) was used to construct the libraries. Then libraries were subjected to paired-end sequencing with pair-end 150-base pair reading length on an Illumina NovaSeq sequencer (Illumina).

For data analysis, the genome of human genome version of hg38 was used as a reference. The sequencing quality was assessed with FastQC (28) and the clean reads were aligned to the reference genome using HISAT2 (29) with default parameters. DESeq2 (30) was used to analyze the DEGs (differently expressed genes) between samples. Parameters for classifying significantly DEGs are ≥2-fold differences (|log2FC|≥1, FC: the fold change of expressions) in the transcript abundance and adjusted p ≤ 0.01. Kyoto Encyclopedia of Genes and Genomes (KEGG) pathway analysis was performed using the Clusterprofile package (26).

### Detection of cytokine production

THP-1 monocytes were seeded at a density of 1 × 10^6^ cells per mL in 12-well plates. Then *E. coli* BEVs at a protein concentration of 1μg/mL and *S.aureus* BEVs at a concentration of 10 μg/mL were added to each well followed by incubation for 6 h. Cells stimulated with PBS served as the negative control.

To measure gene expression of IL-6, IL-8, and IL-1β, total RNA was isolated with an RNA Extraction kit (Tiangen, China) according to the manufacturer’s instructions. 1 μg of isolated RNA was reverse transcribed into cDNA using the 1st Strand cDNA Synthesis SuperMix Kit (Novoprotein, China). Then, 0.5 μl of cDNA was used as the template for each real-time PCR. Real-time PCR was performed using a SYBR qPCR SuperMix (Novoprotein, China) on ABI7500 real-time PCR system (Thermo Fisher Scientific, USA). Primers for target genes are presented in **Supplementary Table 1**. Gene expression was normalized to GAPDH production in each sample, and the fold induction was determined by using the △△C_T_ method. Each experiment was performed with triplicate samples and repeated three times.

To determine the production of cytokines, cell culture supernatants were collected. The levels of IL-8 and IL-1β were measured by enzyme-linked immunosorbent assay kits (ELISA, solarbio, China) according to the manufacturer’s instructions. Each experiment was performed with triplicate samples and repeated three times.

### Statistical analysis

Analysis of cell viability, BEV internalization and cytokine production was performed at least three times independently, and the data are shown as means ± SEM. One-way analysis of variance (ANOVA) using Tukey’s multiple-comparison test was applied to differentiate between groups. Statistical analyses were performed using R software 4.0.2 (R Foundation for Statistical Computing, Austria).

## Results

### Treatment of bacterial culture medium using ϵ-PL could enrich membrane-bound structures

The zeta potentials of BEVs derived from Gram-negative and Gram-positive bacteria are negative owing to a high percentage of negatively charged phospholipids in bacterial membranes. We isolated BEVs from the culture supernatant of *Escherichia coli* (*E. coli*) and *Staphylococcus aureus* (*S. aureus*) by ultracentrifugation. And we confirmed the surface zeta potential values were highly negative (**Supplementary Figure 1**). Epsilon-poly-L–lysine (ϵ-PL) is a hydrophilic linear homo-poly-amino acid, which typically consists of 25 to 35 L–lysine residues. Because of its amino groups, ϵ-PL contains a strong positive charge and can interact with both gram-positive and gram-negative bacteria through electrostatic adsorption.

**Figure 1:**
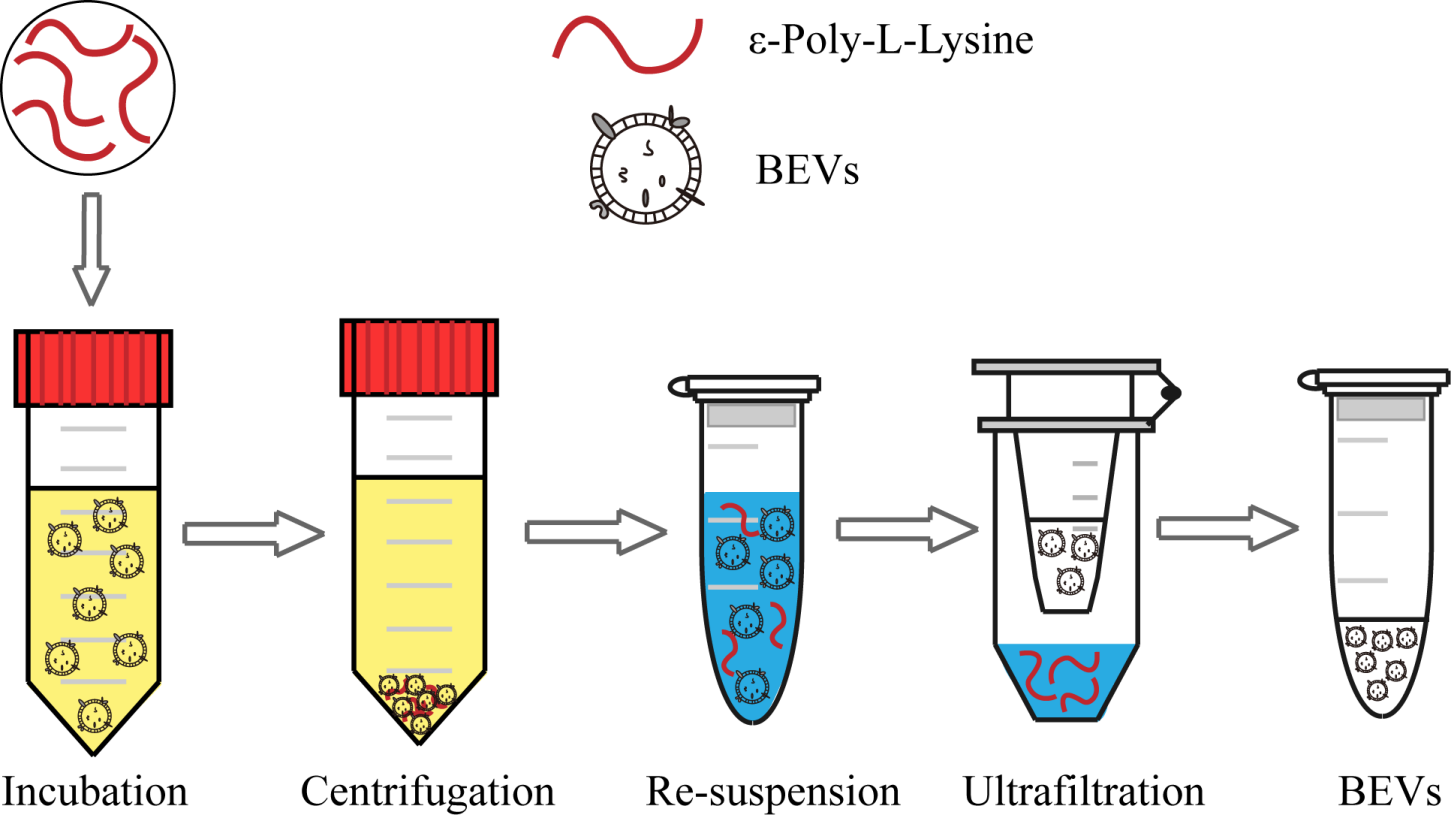
Schematic of the BEV isolation from the bacterial culture medium using the ϵ-PL-based method.

The scheme of the whole isolation process is presented in **Figure 1**. Negatively charged BEVs can bind to positively charged ϵ-PL, which leads to aggregation of BEVs and makes isolation by centrifugation easier. Pellets are re-suspended in a buffer with a high pH value and high concentration of salt. The resulting mixtures are loaded into the assembled ultrafiltration column to remove ϵ-PL and achieve buffer exchange. The recovered BEVs could be used for subsequent analyses directly. In this work, we chose *E. coli* and *S. aureus* grown in LB broth to evaluate the ϵ-PL-based isolation method.

**Figure 2:**
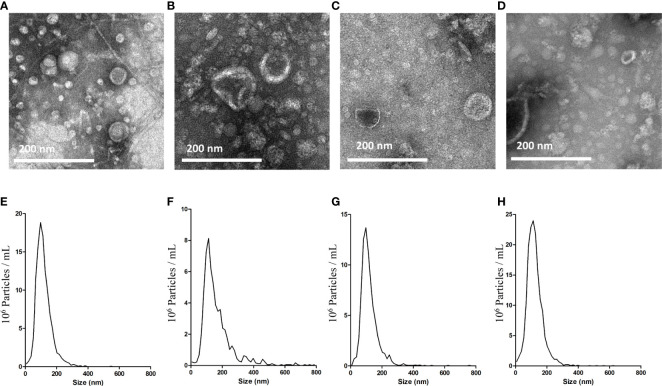
Characterization of BEVs using TEM and NTA. TEM images of BEVs derived from *E. coli* isolated by UC **(A)** or PL **(B)**. TEM images of BEVs derived from *S. aureus* isolated by UC **(C)** or PL **(D)**. Representative particle size-distribution curve of BEVs derived from *E. coli* isolated by UC **(E)** or PL **(F)**. Representative particle size-distribution curve of BEVs derived from *S. aureus* isolated by UC **(G)** or PL **(H)**.

BEVs isolated by the ϵ-PL-based precipitation method (PL) were examined for morphology and size distribution. The results were compared with those of the commonly used ultracentrifugation method (UC). In transmission electron microscopy (TEM) analysis, round particles with typical cup-shaped morphology were identified in all samples (**Figures 2A–D**). We then conducted a Nanoparticle tracking analysis (NTA) to compare the size distributions of the BEVs obtained using the two methods. The majority of vesicles isolated by UC are in the size range of 50–150 nm (**Figures 2E, G**), with a major peak size of around 100 nm. The size distributions of BEVs isolated by PL were comparable to the results of UC, though the average particle size is slightly larger (**Figures 2F, H**).

### Proteomic analysis shows enrichment of vesicle-related proteins for both isolation methods

**Figure 3:**
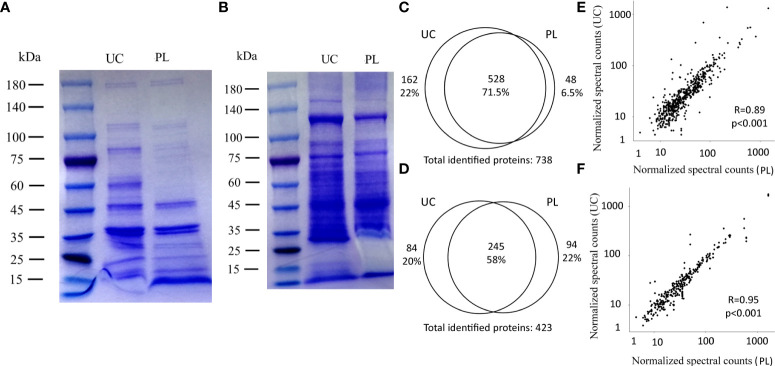
Comparison of identified proteins between UC and PL. SDS-PAGE of BEVs derived from *E. coli* **(A)** and *S. aureus* **(B)** isolated by UC and PL. Venn diagrams showing the identified proteins in *E. coli* **(C)** and *S. aureus* **(D) **BEVs isolated by UC or PL. Correlation analysis of proteins identified in *E. coli* **(E)** and *S. aureus* **(F)** BEVs isolated by UC compared to PL. Shown proteins were identified in at least 2 of the 4 patients in each species.

SDS-PAGE was carried out to evaluate the protein profiles of BEVs isolated by UC or PL. The motion pattern of BEVs in a gel presented similar protein profiles between the two methods (**Figures 3A, B**), although there were several differently represented protein bands (**Figure 3A**). To reveal differences in the protein content in BEVs isolated by different methods, proteomic analysis was performed using liquid chromatography-tandem mass spectroscopy (Data are available *via* ProteomeXchange with identifier PXD034259). As summarized in the Venn diagrams, there were substantial overlaps between the identified proteins in BEVs isolated by the two methods for both *E. coli* and *S. aureus* (**Figures 3C, D**). And the correlations of protein levels between fractions isolated using UC and PL were also high (**Figures 3E, F**). However, some proteins indeed showed differences between the two methods. A representative protein is 60 kDa chaperonin GroEL derived from *E. coli*, which was enriched in the fraction isolated by UC, and may be the reason for the high intensity of the 60-kDa band in the UC group. Although GroEL has been detected in many BEV studies, one study using BEVs purified by density gradient centrifugation and size exclusion chromatography described the protein as a potential contamination marker (31). The implication of GroEL in vesicle biosynthesis requires further in-depth study.

**Figure 4:**
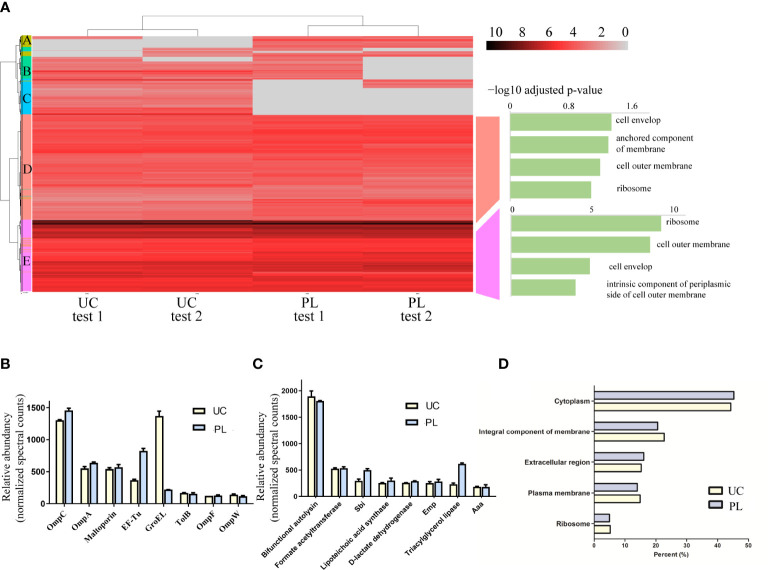
Identification of proteins isolated by the two methods. **(A)** Clustering analysis and subsequent GO enrichment for Cellular Components on the identified common clusters was performed for isolated samples from *E. coli*. The scale of the heatmap shows log2 transformed intensities of the proteins. Absent proteins are displayed in grey. **(B)** The abundance of representative proteins BEVs derived from *E. coli* isolated by UC and PL. **(C)** The abundance of representative proteins BEVs derived from *S. aureus* isolated by UC and PL. **(D)** Distribution of vesicular proteins derived from *S. aureus* based on their subcellular locations.

To determine what type of proteins each method isolates, we employed k-means clustering and visualized the clustering results by heatmap (**Figure 4A**, **Supplementary Figure 2A**, **Supplementary Table 2**). In general, proteins showing similar protein profiles across the two methods were highly abundant (**Figure 4A**, **Supplementary Figure 2** cluster D, cluster E), while the proteins showing differences between the UC and PL groups were frequently low abundant (**Figure 4A**, **Supplementary Figure 2A** cluster A-C). For proteins derived from *E. coli*, we then performed functional enrichment analysis to identify enriched cellular components of the common proteins. The GO annotation of cluster D and E showed enrichment of terms such as “outer membrane” and “ribosome”, supporting the outer membrane vesicle origin of these proteins. And several potential BEV-related proteins (32, 33) were among the most abundant proteins, such as outer membrane porin C (OmpC), outer membrane protein A (OmpA) and elongation factor Tu (EF-Tu) (**Figure 4B**). We also compared the identified proteins with a public dataset (top 50 proteins that are most frequently identified in Gram-negative bacterial outer membrane proteins, **Supplementary Table 3**). Of the top 50 most reported proteins (32), 43 were found in our dataset. Among them, 33 proteins were included in cluster E and 8 were included in cluster D. In contrast, the proteins showing differences between the UC and PL groups (cluster A-C) revealed no significant enrichment in any GO cellular component category, though many of them are categorized as cytosolic protein. Whether these proteins are exported inside BEVs or co-precipitated with the vesicle remains to be validated. For proteins derived from *S. aureus* (**Figure 4C**), the high abundant cluster D consists of proteins involved in cell wall biosynthesis/degradation (bifunctional autolysin, lipoteichoic acid synthase), adhesins (extracellular matrix protein-binding adhesin Emp, and autolysin/adhesin Aaa), metabolic enzymes (triacylglycerol lipase, D-lactate dehydrogenase, and Formate acetyltransferase), and immune evasion factors (immunoglobulin-binding protein Sbi). Most proteins were located in the cytoplasm and membrane (**Figure 4D**), which agreed with what was described earlier (31). Overall, these data indicate that PL isolates a similar vesicle-rich fraction as the commonly used UC method.

### BEVs isolated by PL can be internalized by human monocytic THP-1 cells

**Figure 5:**
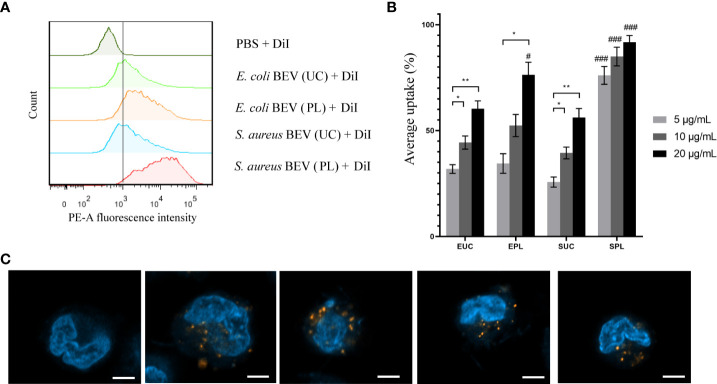
BEVs show successful uptake within THP-1 cells. **(A)** The phycoerythrin (PE-A) channel fluorescence intensity after incubation of cells with DiI-labeled BEVs in a flow cytometry measurement. The dashed line separates PE-A channel positive and negative cells. **(B)** Average percentage uptake of fluorescently labeled BEVs at different concentrations (5, 10 and 20 μg/mL) inside THP-1 cells. EUC, *E. coli* BEV isolated by UC; EPL, *E. coli* BEV isolated by PL; SUC, *S. aureus* BEV isolated by UC; SPL, *S. aureus* BEV isolated by PL. Significant differences between different BEV concentrations are indicated by asterisks: *p < 0.05; **<0.01. Significant differences compared BEVs isolated by different method among the same concentrations are indicated by hash sign: # p < 0.05; ### p < 0.001. **(C)** Confocal microscopy images of internalization of BEVs into THP-1 cells. PBS-treated THP-1 cells served as the negative control. (Left to right: negative control, BEVs derived from *E. coli* isolated by UC and PL, and BEVs derived from *S.aureus* isolated by UC and PL.) Red, DiI-stained BEVs; blue, DAPI-stained nucleus of THP-1 cells. Scale bar: 5 μm.

To find out whether the ϵ-PL-based method impacts the biological activity of BEVs, we verified the ability of BEVs collected by UC or PL to be taken up by recipient cells. It has been reported that BEVs isolated by UC can be internalized by mammalian cells after 4 h of incubation. So, THP-1, a human monocytic cell line, was incubated with DiI-stained BEVs at protein concentrations of 5, 10, and 20 μg/mL. After 4 h of incubation, THP-1 exhibited uptake of BEVs isolated by both UC and PL, as indicated by a shift of fluorescence intensity of cell populations in flow cytometry (**Figure 5A**). A dose-dependent increase in uptake rate was detected for both UC and PL BEVs. (**Figure 5B**) BEVs enriched using ϵ-PL were taken up as well as, or better than those enriched using UC. We validated the flow cytometry results by confocal laser scanning microscopy (**Figure 5C**). Both types of BEVs were uptake by the THP-1 cells, which were confirmed by the appearance of numerous red dots of DiI-stained BEVs. No DiI staining was observed in the negative control group.

### Transcriptional analysis shows triggered immune response in THP-1 cell by PL isolated BEVs

**Figure 6:**
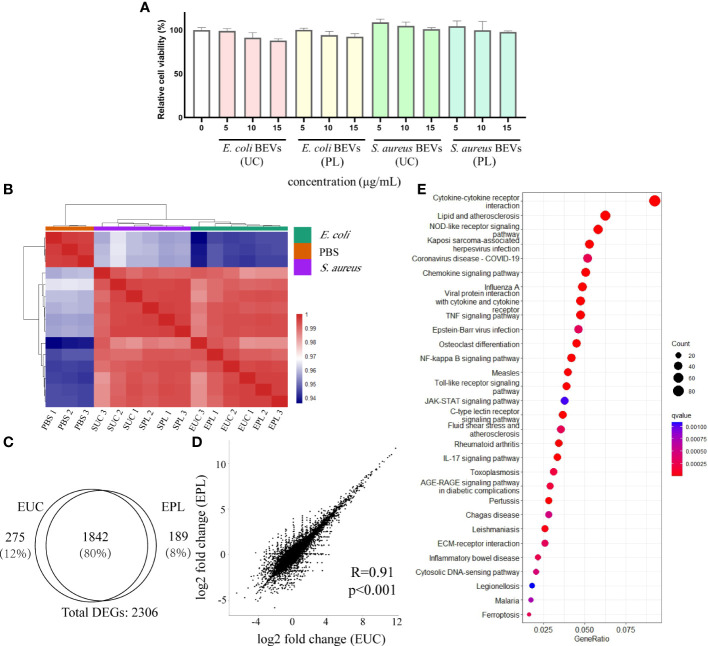
Transcriptional analysis of THP-1 cells stimulated with BEVs. **(A)** Calculated percentage cytotoxicity of cells (EUC, 1 μg/mL *E. coli* BEV isolated by UC; EPL, 1 μg/mL *E. coli* BEV isolated by PL; SUC, 10 μg/mL *S. aureus* BEV isolated by UC; SPL, 10 μg/mL *S. aureus* BEV isolated by PL). **(B)** The heat map was performed by using the Euclidean distance method with complete linkage for all samples (PBS1, PBS2, PBS3 are triplicates of non-stimulated THP-1 cells; EUC1-3 and EPL 1-3 are triplicates of cells stimulated with 1 μg/mL *E. coli* BEV isolated by UC and PL, respectively; SUC1-3 and SPL 1-3 are triplicates of cells stimulated with 10 μg/mL *S. aureus* BEV isolated by UC and PL, respectively). **(C)** Venn diagram of genes that are differentially expressed compared to non-stimulated cells in EUC and EPL group. **(D)** Correlation analysis of DEGs identified in EUC and EPL group. **(E)** Enrichment analysis of KEGG pathways enriched for overlapping DEGs in EUC and EPL group. Top 30 enriched KEGG pathways were selected for visualization.

Examination of whether the BEVs isolated by UC or PL have any cytotoxic effects was performed on THP-1 cells. Various concentrations (0, 5, 10, and 15 μg/mL) of BEVs were applied to THP-1 cells, and the cell line exhibited tolerance to the BEV from all samples during the incubation time (**Figure 6A**). The cells incubated with BEVs showed comparable viability to the positive control group (PBS treated), indicating the BEVs isolated by both methods have compatibility with immune cells and do not show obvious cytotoxic effects.

To address whether BEVs isolated by different methods lead to different host responses, we stimulated THP-1 cells with 1 μg/mL *E. coli* BEV or 10 μg/mL *S. aureus* BEVs. The global transcript profiles were evaluated 6 hours after incubation by the RNA-seq technique, and this time period was reasonably selected to collect the transcription profiles at the early stage of stimulation. The heatmap visualized the pairwise correlations between samples and the correlation values were hierarchically clustered. In comparison to non-stimulated THP-1 cells, BEV stimulated cells triggered a striking alteration in the gene expression patterns, as indicated by the clear separation of clusters of unstimulated groups from the BEVs stimulated groups (**Figure 6B**). Hierarchical clustering also segregated the cells stimulated with *E. coli* BEVs from those of stimulated with *S.aureus* BEVs.

Then differential expression analysis was conducted in BEV-stimulated cells compared with non-stimulated cells by DESeq2 package. Genes with adjusted p-value < 0.01 were considered differentially expressed and we then identified differentially expressed genes (DEGs) based on the values of log2 fold-change (|log2 foldchange| > 1). Compared with non-stimulated cells, *E. coli* UC BEV stimulated cells showed 1337 up-regulated genes and 780 down-regulated genes (**Supplementary Figure 3A**). While *E. coli* PL BEV stimulated cells showed 1341 up-regulated genes and 690 down-regulated gene (**Supplementary Figure 3B**). We found a substantial overlap (≈80%) between the DEGs in the two groups (**Figure 6C**) and the correlations of gene expression fold change were also high (**Figure 6D**). In order to understand how the BEV-stimulation translates into physiological functions, we performed pathway analysis on the overlapping DEGs using the KEGG database. The results showed that the DEGs were mainly related to innate immunity response (**Figure 6E**), such as NF-kappa B signaling pathway, Toll-like receptor signaling pathway NOD-like receptor signaling pathway, and C-type lectin receptors signaling pathway (**Supplementary Figures 3C-F**). For BEVs derived from *S.aureus*, the two methods also showed similar effects (**Supplementary Figures 4A-D**), and the common DEGs enriched in KEGG terms related to innate immunity as well (**Supplementary Figures 4E**)

**Figure 7:**
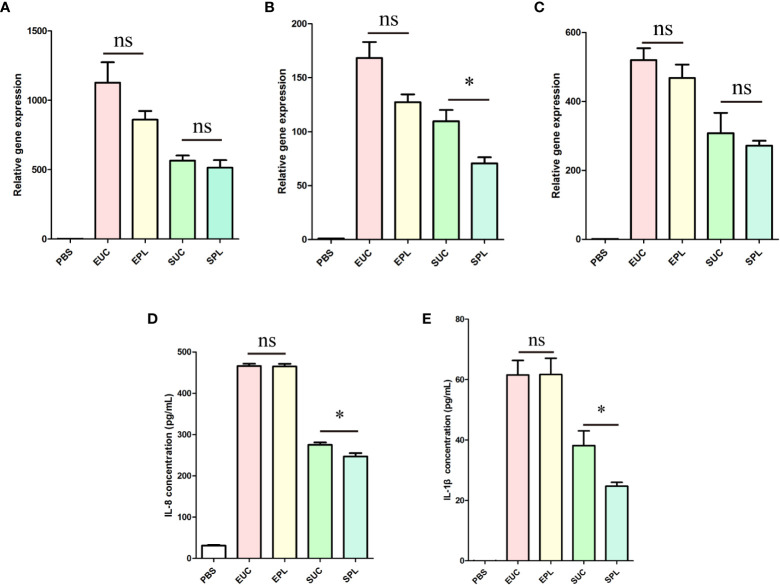
The expression of cytokines by THP-1 cells upon stimulation with BEVs. EUC, 1 μg/mL *E. coli* BEV isolated by UC; EPL, 1 μg/mL *E. coli* BEV isolated by PL; SUC, 10 μg/mL *S. aureus* BEV isolated by UC; SPL, 10 μg/mL *S. aureus* BEV isolated by PL. **(A)** The gene expression of IL-1β. **(B)** The gene expression of IL-6. **(C)** The gene expression of IL-8. **(D)** The release of IL-8. **(E)** The release of IL-1β. n = 3 biological replicates, ± s.e.m. Significant differences compared to the PBS groups are indicated by asterisks: *p < 0.05; ns represents no significance.

To verify the transcriptome analysis data and determine the immunostimulatory activity of the isolated BEVs in host cells, we further investigated the effects of BEVs on the mRNA levels of IL-1β, IL-6, and IL-8 by qRT-PCR. Results showed that the expressions of these pro-inflammatory cytokines were significantly higher (100- to 1000-fold) in all BEV-stimulated groups than in the negative control group (**Figures 7A-C**). THP-1 cells treated with ϵ-PL-only didn’t show significant differences from the PBS group (**Supplementary Figure 5**). Then the overall amounts of secreted IL-1β and IL-8 were analyzed in the culture media of the different experimental groups. BEVs derived from both species promoted the secretion of cytokines in THP-1 cells **Figures 7D, E**). Taken together, these results demonstrated that BEVs enriched by PL have the potential to activate host cells to the same degree as the BEVs isolated by UC.

## Discussion

The production of BEVs is ubiquitously present in Gram-negative and Gram-positive bacteria (34). Recently, BEVs have been considered key players in the exchange of biological signals between microbe and host (5, 35–37). BEVs released by commensal microorganisms have the potential to contribute to host physiology including interaction with immune cells to induce host immunological tolerance or diseases (10, 38), which is an active area of research. However, the isolation processes have been a bottleneck for BEV research due to technical difficulties. There is an urgent need to develop inexpensive, rapid and adaptable methods to enrich BEVs.

Due to the composition characteristics of the phospholipid head groups, the surfaces of bacteria usually contain negative charges (39), thus interacting readily with cationic antimicrobial peptide (40, 41). Extracellular vesicles derived from microbial surfaces also contain excessive negative charges. We analyzed the zeta potential of BEVs derived from *E. coli* (a gram-negative bacterium) and *S. aureus* (a gram-positive bacterium), and the results agreed with what was described earlier (42–44). It has been shown that the adsorption of ϵ-PL, a natural antimicrobial, to the bacterial cell surface plays an important role in its antibacterial activity. While ϵ-PL has been used to develop a broad-spectrum bacterial cell capture technique (23), there is no study for the isolation of BEVs using ϵ-PL. Here we developed a novel ϵ-PL-based isolation method (PL) for rapid enrichment of BEVs from bacterial culture medium. We then demonstrated that BEVs isolated by PL are comparable to those isolated by UC in size distribution, morphology, protein profile and biological functions. The current method is simple, cost-effective and scalable, and is expected to be applied to basic research that requires the isolation of BEVs.

The images of the BEVs enriched by UC and PL were obtained by a TEM for physical characterization. The integrity of the vesicles from all samples was demonstrated by the presence of typical membrane-bound structures. However, we detected a broader particle size range of the BEVs isolated by PL measured by NTA. One reason for this difference could be the aggregation of vesicles caused by the ϵ-PL treatment (21). Further optimization of the reagents and the isolation workflows is necessary.

In terms of identified proteins and associated gene ontologies, the proteome of BEVs isolated by PL is largely comparable to that of BEVs isolated by UC. The correlation of protein levels was high and the predicted localization of proteins is similar between the two methods, with a high abundance of outer membrane protein (*E. coli*) or cytoplasmic protein (*S. aureus*). We then examined the overlap between the proteins derived from *E. coli* with a dataset in EVpedia (top 50 proteins that are most frequently identified in Gram-negative bacterial outer membrane vesicles). The majority of overlapping proteins were found in cluster D and E. Therefore, many of the proteins in these two clusters were able to be confirmed in previous studies on the BEV proteome. For example, the most abundant proteins identified in the samples derived from *E. coli* were outer membrane proteins (OmpC, OmpA, OmpF, etc.) (33) In contrast, the abundant of proteins in cluster A-C were relatively low and many were located in cytosol and plasma membrane. The characteristic in common for this set of proteins is their engagement in metabolic processes, such as nucleotide metabolic in PL-enriched group (cluster A) and amino acid and polysaccharide metabolic in UC-enriched groups (cluster B and C). However, these proteins were normally not considered as typical BEV proteins. Whether these proteins are actually co-isolated contaminants or proteins that are specific to certain subtypes of BEVs remains to be elucidated. It is worth noticing that we identified a protein that was consistently under-represented in *E. coli* BEVs isolated by PL. This protein is GroEL, which is a chaperonin required for protein folding. Interestingly, a previous study (45) showed that GroEL was detected at higher levels in the crude input *E. coli* BEVs than the purified BEVs. The PL method may help to remove a number of contaminating proteins. Future researches are needed to validate the localization of this protein.

The study of the bioactivity of BEVs in cell cultures has gained popularity in an effort to understand their biological functions. The BEVs isolated by UC have been widely used in the studies of interkingdom communication and innate immunity (27, 46–50). As a human monocytic cell line, THP-1 has been extensively used in these BEV exposure studies. For example, a previous study (49) treated THP-1 cells with *Filifactor alocis* BEVs for 24 h and analyzed cytokines in the culture supernatants. They detected various pro-inflammatory cytokines related to inducing immune cell activation and infiltration. Another study (51) stimulated THP-1 cells with BEVs isolated from dust samples for 5 h and found that BEVs increased inflammatory mediators in an NF-κB-dependent manner. THP-1 monocytes have the ability to respond fast to inflammatory activators. The over-expression of inflammation-related cytokines could be detected within several hours of incubation (52). Thus, here we chose the THP-1 monocyte as a model to investigate the immune-modulating effects of the BEVs isolated by different methods.

First of all, we studied the internalization of BEVs into THP-1 cells. After 4 h incubation, THP-1 exhibited uptake of BEVs isolated by both UC and PL. Interestingly, BEVs isolated by PL were taken up better than those enriched by UC. Cationic polymers (i.e., ϵ-PL) and negatively charged molecules can form complexes in aqueous physiological solutions. And it has been widely accepted that these complexes can be internalized by various endocytic routes (53). So, we hypothesized that residual ϵ-PL might improve the internalization of BEVs. The amount of residual ϵ-PL was estimated by SDS-PAGE. For BEVs with a protein content of 20 μg, the residual amount of ϵ-PL was less than 2 μg [< 10%, **Supplementary Figure 6A**)]. Next, ϵ-PL was added into UC BEVs, and the uptake rate was measured by flow cytometry. At the adding amount of 10%, the difference of fluorescence intensity was not as significant as that between BEVs isolated by UC and PL (**Supplementary Figure 6B**), and an obvious shift in the fluorescence profile was only observed when the amount of ϵ-PL was high (**Supplementary Figures 6C, D**). Since the amount of residual ϵ-PL itself was insufficient to enhance endocytosis, the ϵ-PL-based isolation method may affect other biological structures of BEV, and the mechanisms of controlling BEV uptake needs to be further studied.

BEVs display multiple PAMPs and deliver cargos to target host cells. THP-1 cells stimulated with BEVs could undergo substantial reprogramming of their transcriptome. We used RNA sequencing to analyze the changes in gene expression profiles of THP-1 cells by BEV stimulation. The result showed that BEV stimulation triggered a striking alteration in the gene expression patterns. In the meantime, the samples treated with the same species of BEVs grouped together, which indicates the biological functions of PL BEVs are similar to those of UC BEVs. We also found that the response of cells to *E. coli* and *S. aureus* BEVs involved the upregulation of a common set of genes (supp Table), which were enriched in pathways like NF-kappa B signaling pathway, and Toll-like receptor signaling pathway. Similar to what was described in previous studies (46, 47, 54), the common cellular response mainly involved genes related to the immune response, especially those encoding cytokines and chemokines. The results support the concept that BEVs are potent stimulators of innate immune responses. QRT-PCR and ELISA were used to detect the expression of cytokines, and the intact biological function of BEVs isolated by PL was validated. In a word, these results demonstrated that BEVs isolated by our method still retained the *in vitro* biological activity.

In comparison with the isolation method based on UC, the ϵ-PL-based method has the merits of operational simplicity, accessibility and low cost. However, our study still has some limitations. Firstly, the current study was limited to BEVs derived from *E. coli* and *S. aureus*. Evaluation of the isolation results using other bacterial species would lend further support to the efficiency of the ϵ-PL-based technique. Secondly, the components of some nutrient-rich broths may show interference effects against the isolation method (20). Here we mainly tested bacteria grown in the commonly used LB broth, further optimizations of the workflows for different broths are necessary. Thirdly, we only assessed the protein profiles of the isolated fractions. A comprehensive analysis of proteins, nuclear acids, lipids and other metabolites harbored by BEVs could provide more information. Finally, the ultrafiltration step requires a lot of manual operation and can reduce the recovery rate due to the clogging and trapping of BEVs in the filters (55). The use of microfluidics in combination with ϵ-PL is promising in the development of more adaptable separation techniques (56, 57).

In conclusion, we have established an ϵ-PL-based isolation method and demonstrated that it could be used for efficient isolation of BEVs from bacterial culture medium. The new method could contribute to studies on BEVs, including research on the composition of BEVs and interkingdom communication between microbiota and the host.

## Data availability statement

The datasets presented in this study can be found in online repositories. The names of the repository/repositories and accession number(s) can be found below: The proteomics data have been deposited in the ProteomeXchange *via* the PRIDE repository with the data set identifier PXD034259. RNA-seq data were deposited in the Gene Expression Omnibus (GEO) database under the accession number GSE207009.

## Author contributions

SW and DJ conceived and designed the experiments. SW and DJ performed the experiments. SW wrote the paper. SW, DJ and WX revised the manuscript. All authors contributed to the article and approved the submitted version.

## Funding

This work was supported by the National Key R&D Program of China (Grant No. 2021YFE0109300).

## Acknowledgments

The authors would like to thank Dr. Junge Chen for using the DLS device and thank Fengqiang Cao for using the NTA device. We also thank Xun Xu for her support in capturing confocal microscopy images.

## Conflict of interest

The authors declare that the research was conducted in the absence of any commercial or financial relationships that could be construed as a potential conflict of interest.

## Publisher’s note

All claims expressed in this article are solely those of the authors and do not necessarily represent those of their affiliated organizations, or those of the publisher, the editors and the reviewers. Any product that may be evaluated in this article, or claim that may be made by its manufacturer, is not guaranteed or endorsed by the publisher.
