# Supplementary material for: A rapid method for isolation of bacterial extracellular vesicles from culture media using epsilon-poly-L–lysine that enables immunological function research
Source: Front Immunol. 2022 Aug 12;13:930510. doi: 10.3389/fimmu.2022.930510 (PMC9411643; doi:10.3389/fimmu.2022.930510)
Supplement: Supplementary file 1 [file DataSheet_1.docx]

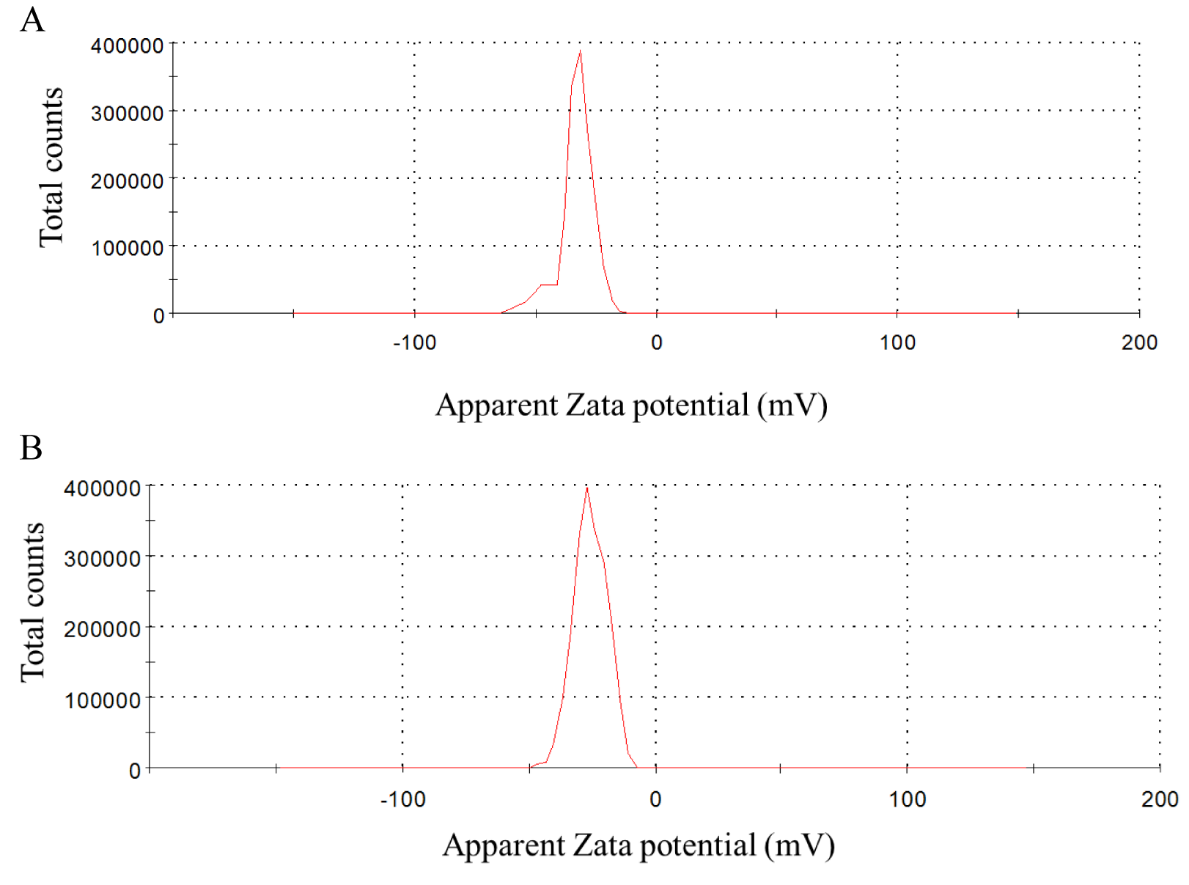


**Supplementary Figure 1.** The surface zeta potential value of BEVs derived from *Escherichia coli* **(A)** and *Staphylococcus aureus* **(B)**.


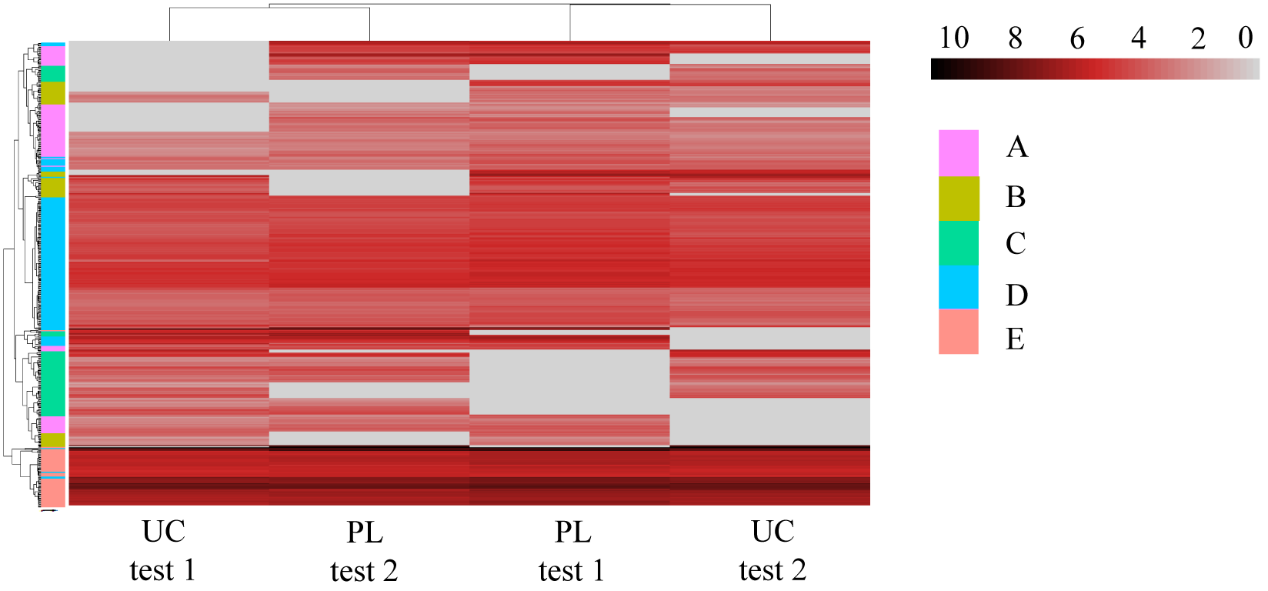


**Supplementary Figure 2.** Clustering analysis on the identified common clusters was performed for isolated samples from *S. aureus*. The scale of the heatmap shows log2 transformed intensities of the proteins. Absent proteins are displayed in grey.


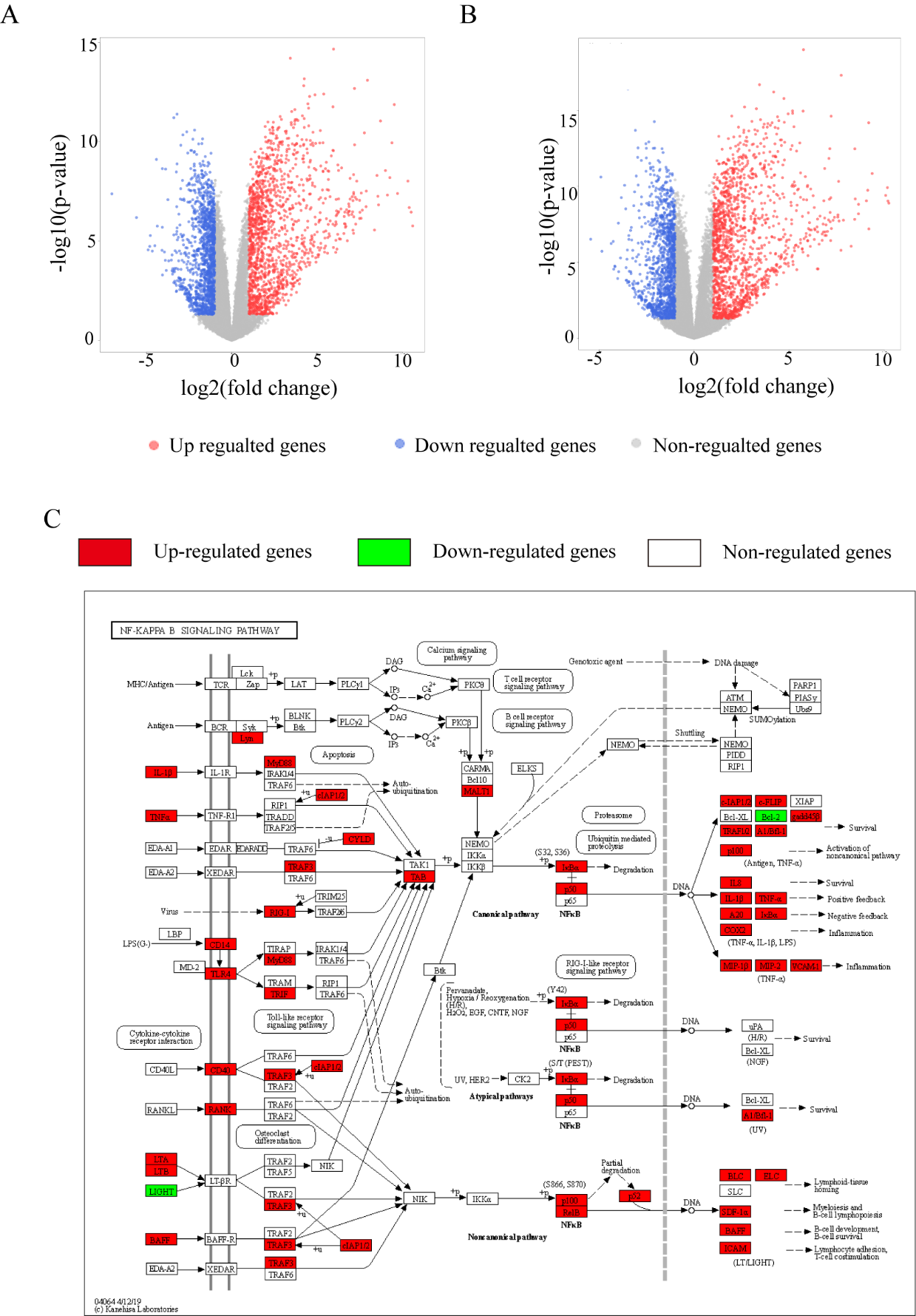


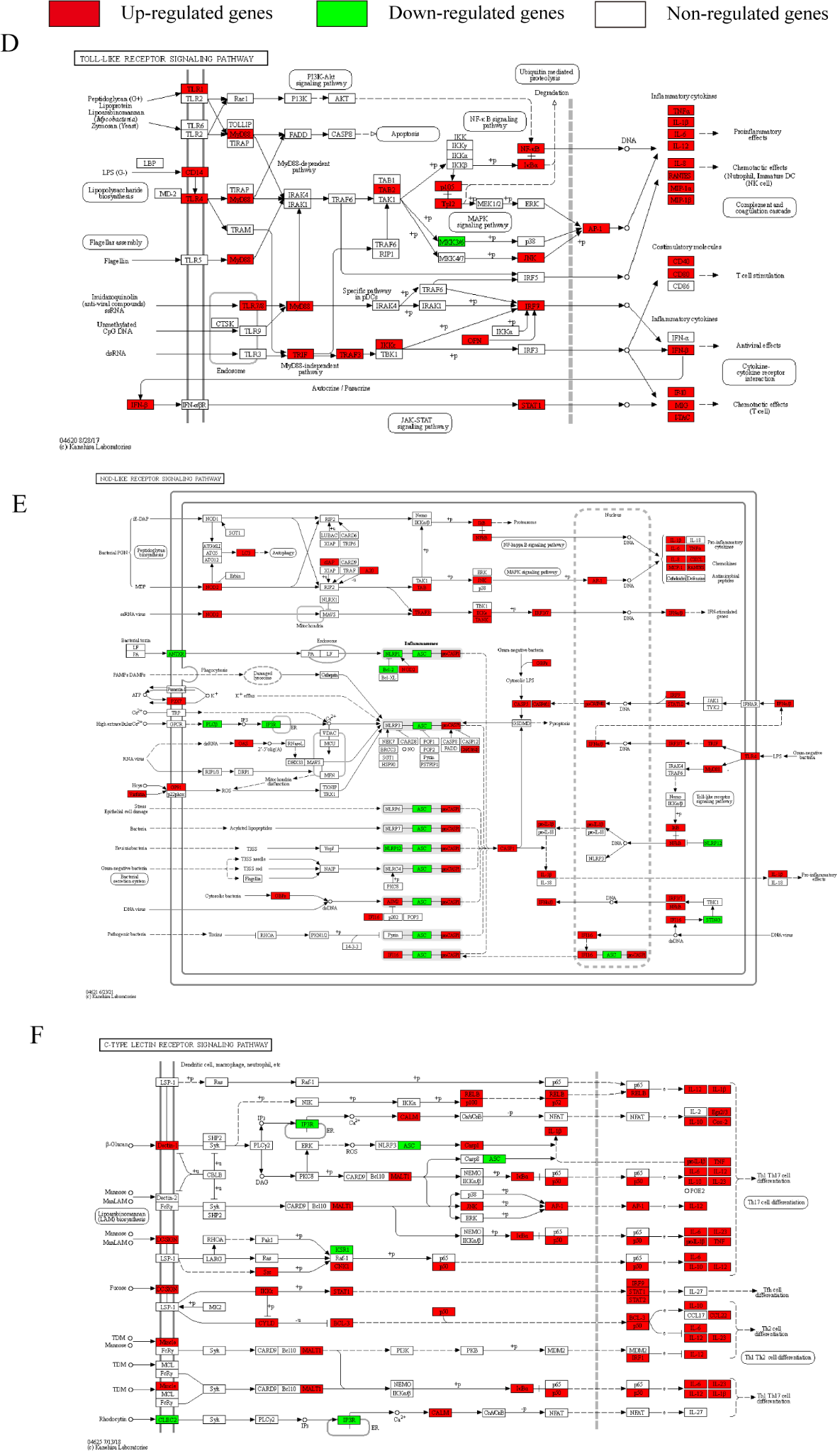


**Supplementary Figure 3. (A)** Volcano plot showing the global transcriptional changes in the THP-1 stimulated with *E. coli* BEVs isolated by UC. **(B)** Volcano plot showing the global transcriptional changes in the THP-1 stimulated with *E. coli* BEVs isolated by PL. **(C-F)** The pathway models of NF-kappa B signaling pathway, Toll-like receptor signaling pathway NOD-like receptor signaling pathway, and C-type lectin receptors signaling pathway were obtained from KEGG Pathway. The red box indicates a gene that is upregulated by BEVs. The green box indicates a gene that is downregulated. The results suggest a strong activation of the innate immunity-related pathway in response to *E. coli* BEVs.


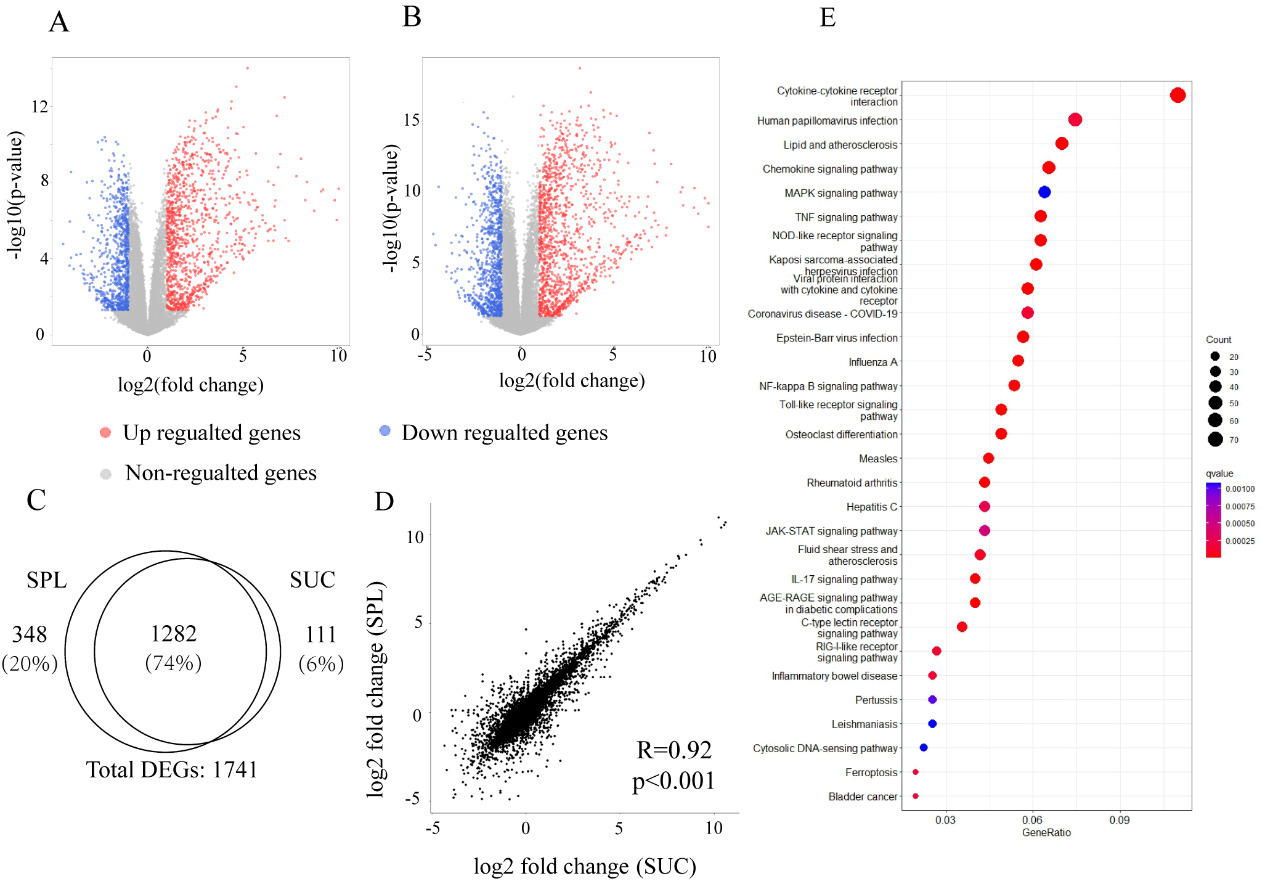


**Supplementary Figure 4. (A)** Volcano plot showing the global transcriptional changes in the THP-1 stimulated with *S. aureus* BEVs isolated by UC. **(B)** Volcano plot showing the global transcriptional changes in the THP-1 stimulated with *S. aureus* BEVs isolated by PL. **(C)** Venn diagram of genes that are differentially expressed compared to non-stimulated cells in SUC (*S. aureus* BEVs isolated by UC) and SPL (*S. aureus* BEVs isolated by PL) groups. **(D)** Correlation analysis of DEGs identified in SUC and SPL groups. **(E)** Enrichment analysis of KEGG pathways enriched for overlapping DEGs in SUC and SPL group. Top 30 enriched KEGG pathways were selected for visualization.


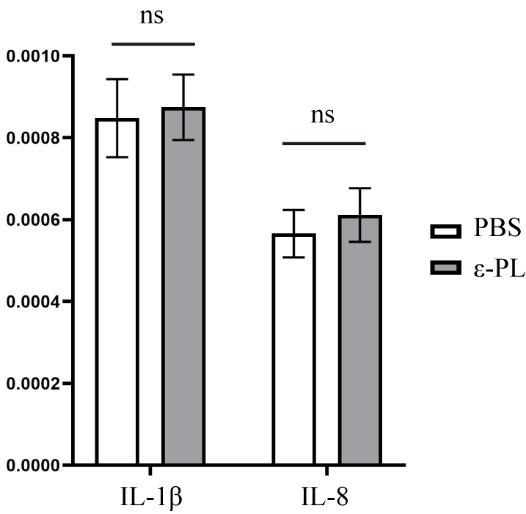


**Supplementary Figure 5.** The gene expression of IL-1β and IL-8 by THP-1 cells upon stimulation with ε-PL. Ns represents no significance.


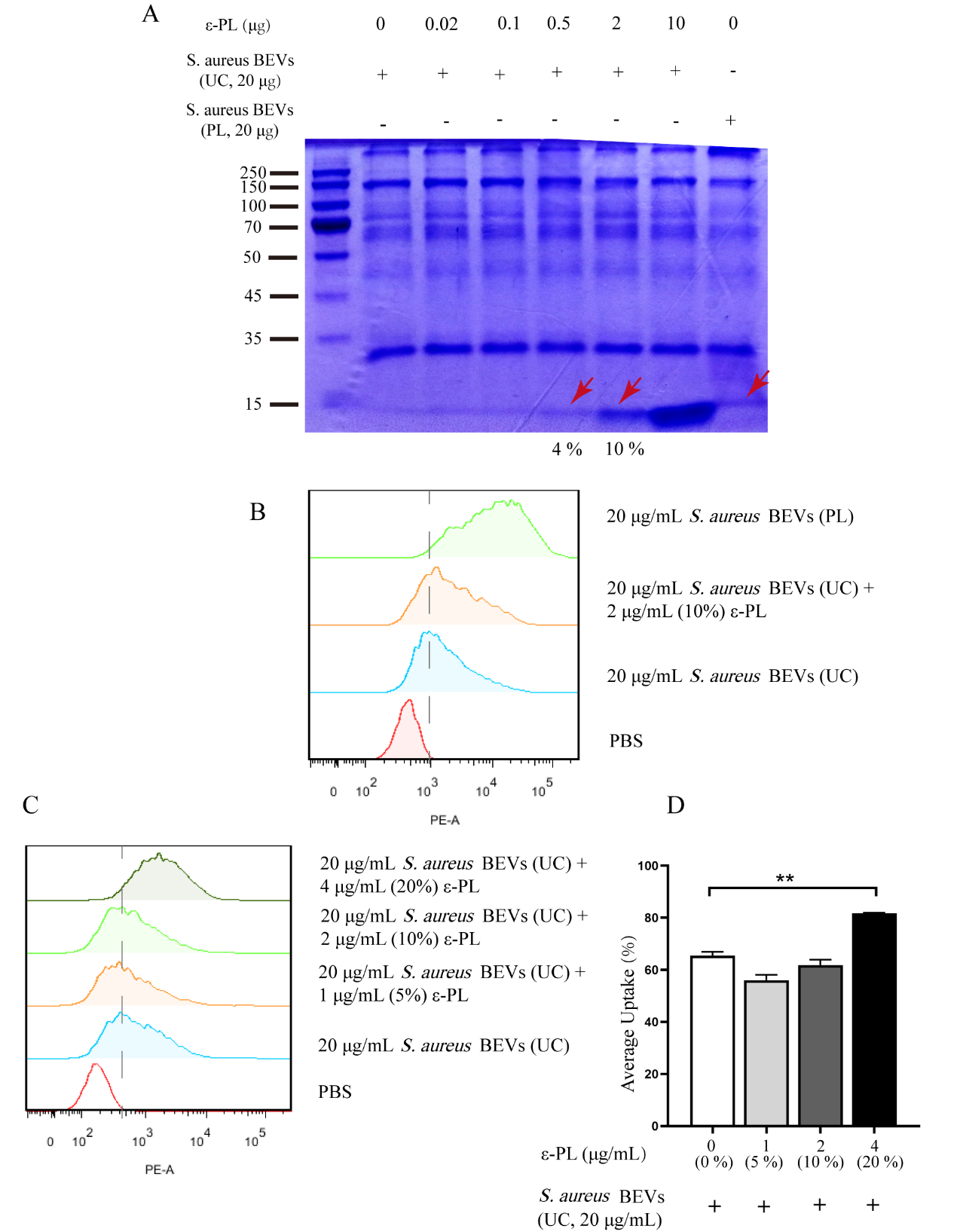


**Supplementary Figure 6. (A)** SDS-PAGE of BEVs derived from *S. aureus* isolated by UC or PL. Different amounts of ε-PL were added into the BEVs isolated by UC. Red arrows highlight the ε-PL band. **(B)** The PE-A channel fluorescence intensity after incubation of cells with DiI-labeled BEVs in a flow cytometry measurement. The dashed line separates PE-A channel positive and negative cells. At the adding amount of 10%, the difference was not as significant as that between BEVs isolated by UC and PL. **(C)** The PE-A channel fluorescence intensity after incubation of cells with DiI-labeled BEVs in a flow cytometry measurement. The dashed line separates PE-A channel positive and negative cells. An obvious shift in the fluorescence profile was only observed when the amount of ε-PL was high. **(D)** Average percentage uptake of fluorescent labeled BEVs added different concentrations (1, 2 and 4 μg/mL) of ε-PL. Significant differences are indicated by asterisks: ** p < 0.01.
